# Symptoms of Prolonged Grief and Self-Reported Health Among Bereaved Family Members of Persons Who Died From Sudden Cardiac Arrest

**DOI:** 10.1177/00302228211018115

**Published:** 2021-05-20

**Authors:** Nina Carlsson, Anette Alvariza, Anders Bremer, Lena Axelsson, Kristofer Årestedt

**Affiliations:** 1 Faculty of Health and Life Sciences, Linnaeus University, Kalmar, Sweden; 2 Department of Internal Medicine, Region Kalmar County, Kalmar, Sweden; 3 Department of Health Care Sciences/Palliative Research Centre, Ersta Sköndal Bräcke University College, Stockholm, Sweden; 4 Capio Palliative Care, Dalen Hospital, Stockholm, Sweden; 5 Department of Ambulance Service, Region Kalmar County, Kalmar, Sweden; 6 Department of Nursing Science, Sophiahemmet University, Stockholm, Sweden; 7 The Research Section, Region Kalmar County, Kalmar, Sweden

**Keywords:** bereavement, family, grief, health, heart arrest, sudden death

## Abstract

Sudden cardiac arrest is common and is one of the leading causes of death in the western world, and the sudden loss following cardiac arrest may have a significant impact on bereaved family members’ health. Therefore, the aim of this study was to describe symptoms of prolonged grief and self-reported health among bereaved family members of persons who died from sudden cardiac arrest, with comparisons between spouses and non-spouses. This was a cross-sectional observation study with 108 adult family members who completed a questionnaire. A fifth of the family members reported prolonged grief, and problems with self-reported health were common, especially regarding anxiety. Spouses reported more problems with prolonged grief and self-reported health compared with non-spouses. The risk of these family members developing prolonged grief and health problems should be recognized, and professional support should be offered.

## Introduction

Death from sudden cardiac arrest is a stressful and challenging situation for family members who are at risk of developing health problems whilst coping with their unexpected bereavement (Bremer et al., 2009; Carlsson et al., 2020; Mayer et al., 2013). The combination of a high incidence and poor survival makes cardiac arrest one of the leading causes of death in many western countries. Even with advancements in cardiopulmonary resuscitation and other treatments, the global mortality is reported to be about 90% when the cardiac arrest takes place outside the hospital (Wong et al., 2019) and about 80% when it occurs in the hospital (Andersen et al., 2019), leaving a large number of family members in grief.

Even though grief is a natural part of life, bereavement is often stressful and may have long-term impact on family members’ lives and perceived health (Shear & Skritskaya, 2012; Stroebe et al., 2007). The specific nature of death due to cardiac arrest, i.e., unexpected, sudden, and often witnessed by family members (Compton et al., 2009), might add to the challenge of bereavement (Kristensen et al., 2012). It is also known that family members can be left with unanswered questions and that circumstances concerning the sudden loss are sometimes obstacles in the process of bereavement (Bremer et al., 2009; Carlsson et al., 2020; Mayer et al., 2013). Coping with bereavement involves oscillating between orientations of loss and restoration, and this oscillation is vital when coping with the changes brought on by the loss and trying to move on with life (Stroebe & Shut, 1999). The absence of such oscillations might be a way to recognise when natural responses to loss instead develop into prolonged grief (Stroebe & Shut, 2010). Prolonged grief disorder implies symptoms lasting more than six months after the loss, and it is characterised by persistent yearning, preoccupation with the deceased, and intense emotional pain that causes significant functional impairment (Prigerson et al., 2009). A close relation to the deceased constitutes a predictive factor for undergoing a prolonged grief process (Kersting et al., 2011; Newson et al., 2011).

Besides prolonged grief, bereavement constitutes a risk for developing health problems, and prior studies have shown increased morbidity and mortality among bereaved family members (Prigerson et al., 1995; Prior et al., 2017; Shah et al., 2013; Stroebe et al., 2007). The risk of developing health problems is found to be higher in the aftermath of unexpected bereavement compared to expected bereavement (Barry et al., 2002; Shah et al., 2013). Health problems may be related to the absence of physical, psychological, and social well-being (WHO, 1948), and examples of physical health problems often reported by bereaved persons are pain, fatigue, heart palpitations, and backaches (Stroebe et al., 2007). Anxiety is an example of a psychological health problem even though it is a natural response to loss (Shear & Skritskaya, 2012). Also, depression (Stroebe et al., 2007) and posttraumatic stress can be consequences of bereavement (Compton et al., 2009). Social well-being (the social dimension of health) refers to the quality and quantity of relationships with family, friends, other significant persons, and wider society. A recent review (Scott et al., 2020) showed that greater informal social support can decrease the risk of developing posttraumatic stress disorder and depressive symptoms in people bereaved by sudden or violent causes of death.

Although most people have the resilience to cope with the loss of a close person (Bonnano, 2009), unexpected bereavement following sudden cardiac arrest creates a potential risk for family members. Therefore, the aim of this study was to describe symptoms of prolonged grief and self-reported health among bereaved family members of persons who died from sudden cardiac arrest, with comparisons between spouses and non-spouses.

## Method

### Design

This cross-sectional observation study was based on questionnaires to bereaved family members six months after the death of a close person following cardiac arrest. The study was approved by the Regional Ethical Review Board in Linköping, Sweden (No. 2017/525-31).

### Participants and Procedure

**Figure 1.**
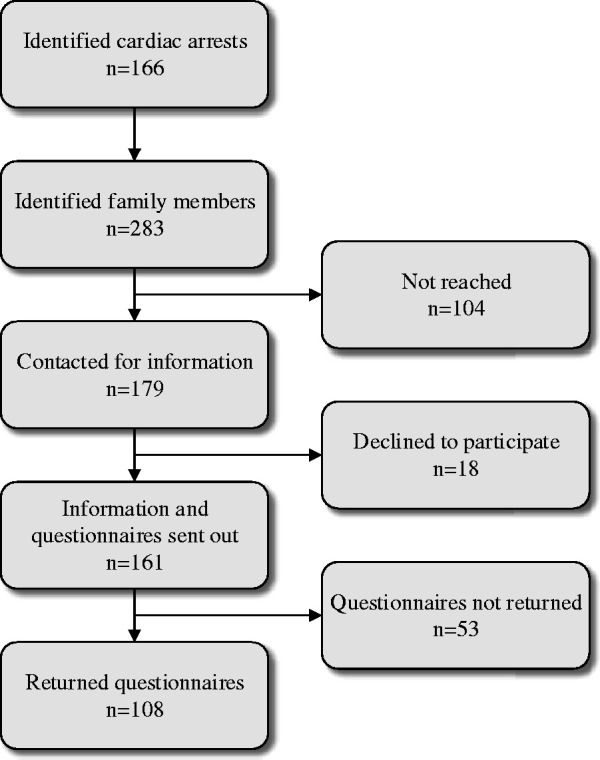
Flowchart Over the Inclusion of Family Members.

Inclusion criteria were a) adult family members (≥18 years) to a person (≥18 years) who had died due to cardiac arrest, b) the cause of the cardiac arrest was heart or lung disease, and c) being able to read and speak Swedish. Family members had been registered in hospital patient record as family members on previous occasions by the person who had died. Family member could therefore also be a friend or other significant person. Hence, family were in this study defined according to Whall’s definition, stating that a family consists of one or more individuals functioning in a way that they perceive to be a family (Whall, 1986). The identification of family members was conducted in two steps. In the first step, the Swedish Register of Cardiopulmonary Resuscitation (https://shlr.registercentrum.se/) was used to identify deceased persons. This screening included one geographic area in south-eastern Sweden and was conducted between September 2018 and June 2020. In total, 166 deceased persons were identified. In the second step, the personal identity number from the Swedish Register of Cardiopulmonary Resuscitation was used to identify registered family members and to obtain their contact information in the hospital patient records. In total, 283 family members were identified and approached by phone by the first author for information and potential inclusion. Three attempts to contact the family members were made, and 179 family members were reached; 161 family members accepted to get information sent to their home, and 18 declined. Family members who accepted to participate were sent a questionnaire and a pre-paid envelope to return the questionnaire to the researchers. In total, 161 questionnaires were sent out, of which 108 were returned (response rate 60%) (Figure 1). No significant differences were detected between the participants and non-participants concerning sex (χ^2^(1) = 1.8, p = 0.178) or relation to the deceased (χ^2^(5) = 10.0, p = 0.075). For ethical reasons, no reminders were sent out. In cases of missing data, family members were contacted by mail and offered the opportunity to complete the questions.

### The Questionnaire

The questionnaire contained demographic questions about the family members and study-specific questions related to the cardiac arrest and to support from the healthcare system. It also contained validated self-reported instruments to assess symptoms of prolonged grief (the Prolonged Grief Disorder) and self-reported health (RAND-36, Health Index, Minimal Insomnia Symptom Scale, Hospital Anxiety and Depression Scale, Posttraumatic Stress Disorder Checklist, and Multidimensional Scale of Perceived Social Support) (Table 1).

**Table 1.** Overview of Self-Reported Instruments.

Instruments	Constructs	Items	Scales	Score range	Cronbach’s α
PG-13	Prolonged grief	13	Total scale	11–55	0.92
RAND-36	General health	2 (36)^a^	Single items	0–4	
HI	Health	9 (10)^b^	Total scale	9–36	0.84
MISS	Insomnia	3	Total scale	0–12	0.81
HADS	Anxiety and depression	14	Two subscales (anxiety and depression)	0–21	0.89, 0.87
PCL-5	Posttraumatic stress	20	Total scale	0–80	0.95
MSPSS	Perceived social support	12	Three subscales (family, friend, and significant others)	1–7	0.92, 0.96, 0.96

PG-13 = Prolonged Grief Disorder-13, HI = Health Index, MISS = Minimal Insomnia Symptom Scale, HADS = Hospital Anxiety and Depression Scale, PCL-5 = PTSD Checklist for DSM-5, MSPSS = Multidimensional Scale of Perceived Social Support.

^a^Only item 1 and 2 concerning general health was used.

^b^Only item 1 to 9 was used as item 10 should not be included to calculate the total scale score (HI index score) according to the constructors of the instrument.

#### Prolonged Grief Disorder-13 (PG-13)

The PG-13 is a development of the Inventory of Complicated Grief (Prigerson et al., 1995), a screening tool for prolonged grief. The PG-13 consists of three parts with a total of 13 items that cover five criteria of prolonged grief (A-E): A is the event criterion (i.e. cardiac arrest), B (items 1 and 2) is related to separation distress, C (item 3) is a duration criterion, D (items 4-12) relates to cognitive, emotional, and behavioural symptoms, and E (item 13) is an impairment criterion. Items 1-5 have the five response options from “*Not at all*” (1) to *“Several times a day”* (5). Items 6-12 have the five response options from “*Not at all”* (1) to *“Overwhelmingly”* (5). Items 3 and 13 are answered with “*Yes”* or “*No”*. A total scale score can be calculated by summing the responses for all items except 3 and 13. The scale score can vary between 11 and 55, with higher scores indicating higher symptom levels of prolonged grief (Prigerson et al., 2009). According to Pohlkamp et al. (2018), a score ≥35 suggests the probable presence of prolonged grief disorder. The instrument has been translated into Swedish and has shown good measurement properties regarding reliability and validity (Pohlkamp et al., 2018). Cronbach’s alpha was 0.92 in the present study.

##### RAND-36

The RAND-36 is constructed to measure health-related quality of life (Hays et al., 1998). This multidimensional instrument includes 36 items, of which 35 cover the following eight health concepts: physical functioning, role physical, role emotional, social function, emotional well-being, energy/fatigue, pain, and general health. In the present study, only two global health questions from RAND-36 were used, one about present health (item 1, referred to as RAND-1) and one about health transition (item 2, referred to as RAND-2). The item about present health (“In general, would you like to say your health is:”) has five response options ranging from *“Excellent”* (1) to *“Poor”* (5) and is included in the dimension “General health”. However, the item is commonly used as a single item to measure self-reported health. The item about health transition (“Compared to one year ago, how would you rate your health in general now?”) also has five response options ranging from “*Much better now than one year ago*” (1) to “*Much worse now than one year ago*” (5) and is not included in the eight health dimensions. The instrument has been translated into Swedish and has shown good measurement properties regarding reliability and validity (Orwelius et al., 2017).

#### Health Index (HI)

The HI was developed by Hansagi and Rosenqvist (unpublished) to measure self-reported health. The HI consists of ten items rated on a four-point response scale (1-4). The initial nine items include energy, temper, fatigue, loneliness, sleep, vertigo, bowel function, aches and pains, and mobility. The responses are summed to an index score that varies from 9 to 36, with higher scores indicating better health. No cut-off scores have been suggested. The HI has shown good measurement properties regarding reliability and validity (Langius et al., 1993; Nordström et al., 1992). Cronbach’s alpha was 0.84 in the present study.

#### Minimal Insomnia Symptom Scale (MISS)

The MISS consists of three items for measuring insomnia. The items are rated on a five-point response scale that varies from “*Not at all”* (0) to “*Extremely”* (4). The total score ranges from 0 to 12. A higher score indicates higher symptom levels, and a cut-off score of ≥6 is recommended to detect probable insomnia. The MISS has shown good measurement properties regarding reliability and validity (Broman et al., 2008). Cronbach’s alpha was 0.81 in the present study.

#### Hospital Anxiety and Depression Scale (HADS)

The HADS was developed to measure symptoms of anxiety and depression. The instrument consists of 14 items and is two-dimensional, i.e., seven items reflect symptoms of anxiety, and seven items reflect symptoms of depression. Each item has four response options ranging from 0 to 3. The items that reflect anxiety and depression respectively are summed to a subscale score, with a possible range between 0 and 21. Higher scores indicate higher symptom levels of anxiety and depression (Zigmond & Snaith, 1983). Cut-off scores have also been suggested as normal range (0–7), suggested presence (8–10), and probable presence (11–21) (Snaith, 2003). The instrument has shown good measurement properties in terms of reliability and validity (Djukanovic et al., 2017; Lisspers et al., 1997). Cronbach’s alpha was 0.89 and 0.87 for items reflecting symptoms of anxiety and depression, respectively.

##### PTSD Checklist for DSM-5 (PCL-5)

The PCL-5 is designed to measure symptom severity of posttraumatic stress according to DSM-5 criteria (Weathers et al., 2013). The instrument consists of 20 items, all responded to on a 5-point Likert-type scale ranging from “*Not at all”* (0) to “*Extremely”* (4). The total score ranges between 0 and 80, with higher scores indicating higher levels of symptom severity. For the present study, a cut-off sore of ≥38 was used, suggesting the probable presence of posttraumatic stress disorder. The instrument has shown good measurement properties regarding reliability and validity (Sveen et al., 2016). Cronbach’s alpha was 0.95 in the present study.

#### Multidimensional Scale of Perceived Social Support (MSPSS)

The MSPSS was developed to measure perceived social support. The instrument consists of 12 items, all rated on a seven-point Likert scale ranging from “*Very strongly disagree”* (1) to “*Very strongly agree”* (7). Three subscale scores can be calculated – perceived social support from family, friends, and significant others. The subscale scores are calculated by dividing the sum score for each subscale by the number of items to give a possible score range between 1 and 7, and higher scores indicate higher levels of perceived social support. No cut-off scores have been suggested (Zimet et al., 1988). The Swedish version has shown good measurement properties in terms of reliability and validity (Ekbäck et al., 2013). In the present study, Cronbach’s alpha was 0.92 for Family, 0.96 for Friends, and 0.96 for Significant others.

### Data Analysis

Missing data not exceeding 25% per scale were replaced using the person mean score. In total, ten missing values from ten participants were replaced. Family members were grouped into spouses and non-spouses. Descriptive statistics were used to present demographic data and study variables. The Mann-Whitney U-test was used to compare symptoms of prolonged grief and self-reported health between spouses and non-spouses. The Wilcoxon signed-rank test was used to make within-group comparisons between HADS anxiety and HADS depression. The significant level was set at p < 0.05. Statistical analyses were performed with Stata/MP 15.1 for Mac (StataCorp LLC, College Station, TX, USA).

## Results

### Characteristics of Participants and the Deceased Persons

The characteristics of the participants (n = 108) are presented in Table 2. Their median age was 61.5 years (range = 25-87 years), and one third (n = 36, 33%) was bereaved spouses of the person who died while two thirds (n = 72, 67%) were non-spouses. A majority were women (n = 74, 69%), were Swedish born (n = 105, 97%), had children (n = 93, 86%), and had a secondary school degree or lower (n = 74, 69%). Most of the 72 non-spouses were adult children of the deceased person (n = 55, 76% [not in the table]), were married (n = 40, 56%), and were cohabiting (n = 59, 82%). All of the bereaved spouses lived alone and were significantly older (Mdn = 72 vs. 55, p < 0.001), were more commonly retired (86% vs. 28%, p < 0.001), and had a lower education (p = 0.004) compared to non-spouses. About one third of all of the participants (n = 35, 32%) had been present at the cardiac arrest and/or the cardiopulmonary resuscitation, and this was more common for spouses than non-spouses (58% vs. 19%) (Table 2).

**Table 2.** Characteristics of Participants.

	All, n=108	Spouse, n=36	Non-spouse, n=72	p-value
Age, Mdn (q1–q3) [range]	61 (51.5–71) [25–87]	71.5 (68–78) [60–87]	55 (48.5–62.5) [25–81]	<0.001^a^
Sex, n (%)				0.884^b^
Woman	74 (69)	25 (69)	49 (68)	
Men	34 (31)	11 (31)	23 (32)	
Country of birth, n (%)				1.000^c^
Sweden	105 (97)	35 (97)	70 (97)	
Other Nordic country	3 (3)	1 (3)	2 (3)	
Marital status, n (%)				n/a
Widow/widower	38 (35)	36 (100)	2 (2)	
Married/registered partner	40 (37)	0 (0)	40 (56)	
Unmarried	17(16)	0 (0)	17 (24)	
Divorced	9 (8)	0 (0)	9 (13)	
Other	4 (4)	0 (0)	4 (6)	
Has children, n (%)				0.077^b^
Yes	93 (86)	34 (94)	59 (82)	
No	15 (14)	2 (6)	13 (18)	
Cohabiting, n (%)				n/a
Living alone	49 (45)	36 (100)	13 (18)	
Living with someone else	59 (55)	0	59 (82)	
Occupation, n (%)				<0.001^c^
Retired	51 (47)	31 (86)	20 (28)	
Employed	47 (44)	5 (14)	42 (58)	
Other	10 (19)	0	10 (14)	
Highest education, n (%)				0.004^b^
Primary school	32 (30)	18 (50)	14 (19)	
Secondary school	42 (39)	9 (25)	33 (46)	
University	32 (30)	8 (22)	24 (33)	
Not stated	2 (2)	1 (3)	1 (1)	
Presence at the CA, n (%)				n/a
FPDR				
Witnessed the CA	14 (13)	8 (22)	6 (8)	
Performed CPR	11 (10)	7 (19)	4 (6)	
Witnessed CA and performed CPR	7 (6)	6 (17)	1 (1)	
Arrived during CPR	3 (3)	0	3 (4)	
No FPDR				
Informed during CPR	7 (6)	3 (8)	4 (6)	
Informed after CPR	66 (61)	12 (33)	54 (75)	

FPDR=Family Presence During Resuscitation.

^a^Mann-Whitney U test.

^b^Chi-square test.

^c^Fisher’s exact test.

Most of the 73 cardiac arrests took place out of hospital (n = 59, 81%), the median age of the deceased persons was 75 years (range = 53–93 years), and 58% (n = 42) were men. A majority of the family members (n = 93, 86%) had not been offered professional support from the healthcare system. Professional support from a counsellor, psychologist, or equivalent after the loss was reported by 15% (n = 16) of the family members.

### Symptoms of Prolonged Grief

The family members reported a median score of 23 (q1–q3 = 16–33) on symptoms of prolonged grief, and spouses reported significantly higher levels compared with non-spouses (p < 0.001). Using the cut-off scores, 29% (n = 10) of the spouses reported the probable presence of prolonged grief in contrast to 13% (n = 9) of non-spouses (Table 3).

**Table 3.** Symptoms of Prolonged Grief and Self-Reported Health in Relation to Spouses and Non-Spouses.

	Valid numbers	All,n=108	Spouses, n = 36	Non-spouses, n = 72	p-value^a^
Symptoms of prolonged grief (PG-13), Mdn (q1–q3)	107	23 (16–33)	29 (21–36)	21 (14–30)	<0.001
No cases (11–34), n (%)		88 (82)	25 (71)	63 (88)	
Cases (35–55), n (%)		19 (18)	10 (29)	9 (13)	
Self-reported health (RAND -36), Mdn (q1–q3)					
General health	108	3 (2–4)	3 (3–4)	3 (2–4)	0.025
General health compared to one year ago	108	3 (3–4)	3 (3–4)	3 (3–4)	0.902
Self-reported health (HI), Mdn (q1–q3)	108	27 (23–29)	25 (21.5–28.5)	27 (24–29.5)	0.118
Energy		3 (2–3)	3 (2–3)	3 (2–3)	0.446
Temper		3 (2–3)	3 (2–3)	3 (2.3–3)	0.306
Tiredness		2 (2–3)	2.3 (2–3)	2 (2–3)	0.917
Loneliness		3 (2–4)	2.5 (2–3)	3 (3–4)	<0.001
Sleep		3 (2–3)	2 (2–3)	3 (2–3)	0.199
Vertigo		3 (3–4)	3 (3–4)	3 (3–4)	0.155
Bowel function		3 (3–4)	3 (3–4)	3 (3–4)	0.854
Pain		3 (2–3)	3 (2–3)	3 (2–3)	0.626
Mobility		3 (3–4)	4 (2.5–4)	3 (3–4)	0.749
Insomnia, (MISS), Mdn (q1–q3)	108	4 (2–6.5)	4.5 (2–6.5)	4 (2–6.5)	0.547
No insomnia (0–6), n (%)		81 (75)	27 (75)	54 (75)	
Insomnia (7–12), n (%)		27 (25)	9 (25)	18 (25)	
Symptoms of anxiety (HADS), Mdn (q1–q3)	107	5 (1–8.5)	5 (2–8.5)	5 (1–9)	0.862
Normal range (0–7), n (%)		75 (70)	25 (71)	50 (69)	
Suggested presence (8–10), n (%)		14 (13)	6 (17)	8 (11)	
Probable presence (11–21), n (%)		18 (17)	4 (11)	14 (19)	
Symptoms of depression (HADS), Mdn (q1–q3)	107	2 (1–7)	3 (1–7)	2 (1–7)	0.297
Normal range (0–7), n (%)		87 (81)	30 (86)	57 (79)	
Suggested presence (8–10), n (%)		13 (12)	2 (6)	11 (16)	
Probable presence (11–21), n (%)		7 (7)	3 (9)	4 (6)	
Symptoms of posttraumatic stress (PCL–5), Mdn (q1–q3)	108	8 (1–20)	15 (4–20)	7 (0–20.5)	0.271
No cases (0–37), n (%)		101 (94)	35 (97)	66 (92)	
Cases (38–80), n (%)		7 (6)	1 (3)	6 (8)	
Social support (MSPSS)					
Family, Mdn (q1–q3)	105	6.8 (5.5–7)	6.8 (5.8–7)	6.8 (5.3–7)	0.478
Low/moderate (1.0–5.0), n (%)		19 (18)	3 (9)	16 (23)	
High (5.1–7.0), n (%)		86 (82)	31 (91)	55 (77)	
Friends, Mdn (q1–q3)	104	6.3 (5–7)	6 (5–6.8)	6.5 (5–7)	0.284
Low/moderate (1.0–5.0), n (%)		31 (30)	9 (27)	22 (31)	
High (5.1–7.0), n (%)		73 (70)	24 (73)	49 (69)	
Significant others, Mdn (q1–q3)	104	6.8 (5.6–7)	6.5 (5.5–7)	7 (5.8–7)	0.116
Low/moderate (1.0–5.0), n (%)		20 (19)	7 ( 21)	13 (18)	
High (5.1–7.0), n (%)		84 (81)	26 (79)	58 (82)	

PG-13  =  Prolonged grief disorder-13, PCL-5  =  PTSD Checklist for DSM-5, HADS  =  Hospital Anxiety and Depression Scale, HI  =  Health Index, MISS  =  Minimal Insomnia Symptom Scale, MSPSS  =  Multidimensional Scale of Perceived Social Support.

^a^Mann-Whitney U test.

**Figure 2.**
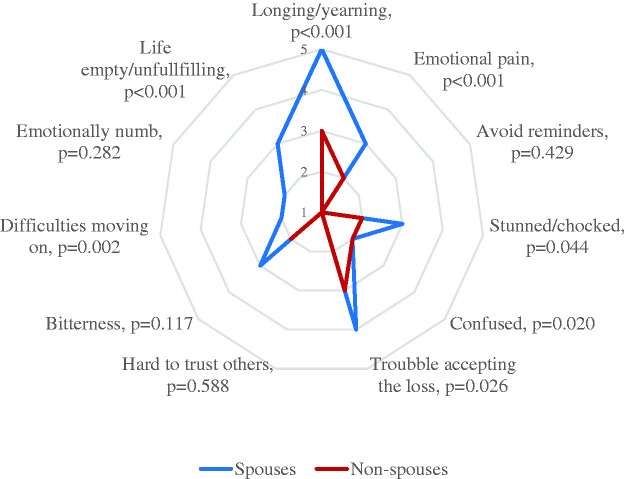
Median Values for the Item Responses on the PG-13 in Spouses and Non-Spouses. Higher values indicate more severe problems (p-values based on Mann-Whitney U test).

On the item level on the PG-13, family members scored highest on having felt longing for the person they lost (Mdn = 4, q1–q3 = 3–5), emotional pain (Mdn = 3, q1–q3 = 1–4), and problems with accepting the loss (Mdn = 3, q1–q3 = 2–4). Lowest scores were reported for avoiding reminders that the person they lost is gone (Mdn = 1, q1–q3 = 1–1), difficulties in trusting others (Mdn = 1, q1–q3 = 1–1), difficulties moving on (Mdn = 1, q1–q3 = 1–2), and emotional numbness (Mdn = 2, q1–q3 = 1–3). Spouses reported significantly higher scores than non-spouses on longing or yearning (p < 0.001), emotional pain (p < 0.001), feeling stunned or chocked (p = 0.044), feeling confused about their role in life (p = 0.020), feeling that life is unfulfilling, empty, or meaningless since the loss (p < 0.001), and having difficulties accepting the loss (p = 0.026) and moving on (p = 0.007) (Figure 2).

### Self–Reported Health

#### HADS

Family members reported significantly higher symptom levels for anxiety than depression (p < 0.001). No significant differences were seen between spouses and non-spouses concerning symptoms of anxiety and/or symptoms of depression. Using the cut-off scores, 28% (n = 10) of the spouses and 31% (n = 22) of the non-spouses reported suggested or probable presence of anxiety. For depression, the corresponding presence was 14% (n = 5) and 21% (n = 15), respectively (Table 3).

#### PCL–5

Family members reported a median score of 8 (q1–q3 = 1–20) on symptoms of posttraumatic stress syndrome. No significant differences were seen between spouses and non-spouses. One of the spouses (3%) and six of the non-spouses (8%) reported the probable presence of post-traumatic stress syndrome (Table 3).

##### RAND-1 and RAND-2

For RAND-1 (i.e., present health), the median rating was “*good health*”, while 25% reported their health as either “*fair*” or “*poor*”. For RAND-2 (i.e., health transition), the median rating was “*about the same health*”, while 25% reported their health as “*somewhat worse*” or “*much worse*” compared to one year ago. Spouses reported significantly poorer present health compared with non-spouses (p = 0.025), but no difference was seen for health transition (Table 3).

#### HI

Self-reported health as measured by the HI was high on average with a median score of 27 (q1–q3 = 23–29). Most reported health problems were regarding tiredness (Mdn = 2, q1–q3 = 2–3), lack of energy (Mdn = 3, q1–q3 = 2–3), temper (Mdn = 3, q1–q3 = 2-3), lack of sleep (Mdn = 3, q1–q3 = 2–3), and pain (Mdn = 3, q1–q3 = 2–3). Spouses reported significantly more problems with loneliness compared with non-spouses (p < 0.001). No other differences were detected between the groups (Table 3).

#### MISS

Family members reported a median score of 4 (q1–q3 = 2–6.5) on insomnia, and no significant differences were seen between spouses and non-spouses. Using the cut-off score, 25% of spouses (n = 9) and 25% of non-spouses (n = 18) reported the probable presence of insomnia (Table 3).

#### MSPSS

High levels of social support were reported for all sources of support by the non-spouses: Family (Mdn = 6.8, q1–q3 = 5.5–7), Friends (Mdn = 6.3, q1–q3 = 5–7), and Significant others (Mdn = 6.8, q1–q3 = 5.6–7). No significant differences were seen between spouses and non-spouses (Table 3).

## Discussion

To our best knowledge this is one of the first studies to describe symptoms of prolonged grief and self-reported health among bereaved family members following the death of a close person from sudden cardiac arrest. Overall, symptoms of prolonged grief and self-reported health problems were common. Furthermore, spouses reported more problems with symptoms of prolonged grief and self-reported health than non-spouses.

In the present study, about 1 in 5 family members reported symptoms of prolonged grief, which is twice as many as reported in a meta-analysis of a bereaved adult population who suffered the loss of a loved one due to non-violent death (Lundoff et al., 2017). It could be that the higher prevalence in the present study is related to the sudden loss and unexpected bereavement. This assumption is partly supported by another meta-analysis of prolonged grief following violent deaths, such as accidents, disasters, or terrorism, which concluded that nearly half of the bereaved adults experienced prolonged grief (Djelantik et al., 2020). Thus, one could argue that deaths by sudden cardiac arrest have similarities with violent deaths in terms of the grief that afflicts the bereaved. In comparison with the results in the present study, there is reason to believe that the suddenness of a death, and not just the cause of the death, can have a significant impact on prolonged grief. According to a review by Kristensen et al. (2012), a combination of suddenness and violence often causes shock among family members making the loss hard to grasp. Therefore, the grief process might take longer and grief reactions might intensify as the initial shock gradually wears off.

The prevalence of prolonged grief was significantly more common in spouses compared with non-spouses. In fact, the proportion of spouses who reported symptoms of prolonged grief in the present study was similar to the proportion of bereaved parents who experienced prolonged grief after the loss of a child (Pohlkamp et al., 2018). Despite the fact that loss of a loved one is an individual experience and cannot be compared, a review based on 42 studies (Morris et al., 2019) concluded that the loss of a child represents a certain level of severity and might be seen as a subgroup in bereavement research. Thus, the present results suggest that severe symptoms of prolonged grief are also common in bereaved spouses after death by sudden cardiac arrest, which raises the question of whether this group should also be considered a subgroup in bereavement research.

One in four family members in the present study reported their general health as fair or poor and their health to be worse compared to a year ago. Health problems such as tiredness and sleeping problems were commonly reported, and one fourth met the criterion for insomnia. There is a growing body of evidence showing that sleep problems and insomnia are associated with psychological symptoms in bereavement, and sleep disturbances can lead to an increased risk for a vicious circle of grief reactions (Pohlkamp et al., 2019), anxiety, and depression (Alvaro et al., 2013).

Psychological health problems in terms of anxiety and depression were common in the present study, both in spouses and non-spouses. The high share of bereaved family members with psychological health problem, particularly anxiety, might be related to the fact that sudden death by cardiac arrest serves as a reminder of one’s own and others’ mortality. Additionally, the loss may generate fear of suffering a cardiac arrest oneself (Carlsson et al., 2020; Yeates et al., 2013). A review by Shear and Skritskaya (2012) argues that confrontation with one’s own death is a natural trigger of anxiety, and although bereavement anxiety is a normal response to loss, it may disturb the grief process and cause prolonged grief (Shear & Skritskaya, 2012). Prolonged grief, anxiety, and depression may be hard to separate because they have a high degree of symptom overlap. For example, about half of those diagnosed with anxiety or depression have been shown to have one or more additional affective/mood diagnoses (Brown & Barlow, 2009). However, yearning and longing for the deceased person are symptoms of prolonged grief that are not usually seen in depression (Shear, 2012). In addition, grief is always preceded by a loss, which is in contrast to depression (Wijngaards-de Meij et al., 2005). Sudden and unexpected bereavement may additionally generate symptoms of posttraumatic stress and anxiety that can co-exist with prolonged grief and worsen the symptoms (Shear, 2012).

The bereaved family members reported only minor problems with posttraumatic stress. This finding was unexpected because a third of the bereaved family members had been present, witnessed, and/or performed resuscitation attempts, which is known to be associated with posttraumatic stress disorder (Compton et al., 2009). Family members who are present during the cardiac arrest also risk witnessing futile or ritualistic use of resuscitation. Such use is based on the healthcare professionals’ assumption that resuscitation attempts *per se* would benefit the family members, but instead family members risk being deprived of support and acute grief counselling at the scene (Bremer & Sandman, 2011). In contrast, providing emotional and psychological support to family members during resuscitation might reduce stress and prevent symptoms of posttraumatic stress disorder, anxiety, and depression (Soleimanpour et al., 2017).

Even though most of the bereaved family members reported high levels of social support, experiences of loneliness were common. In addition, spouses reported significantly more problems with loneliness compared to non-spouses. It is well known that loss of a relationship can cause deep feelings of loneliness even when having other close relationships (Dahlberg, 2007), and experiences of loneliness also need to be acknowledged because they are a risk factor for serious medical conditions such as coronary heart diseases and stroke (Valtorta et al., 2016). In addition, reduced social contacts have been found to have profound effects on all types of mortality (Holt-Lunstad et al., 2010). Thus, relationships with other people appear to be a fundamental part of human existence and therefore closely connected to the experience of health.

Finally, a majority of the family members in the present study were not offered any kind of support from the healthcare services at the time of their sudden loss. The European Resuscitation Council (ERC) argues that healthcare professionals should receive training in supportive care and end-of-life decisions and in how to communicate these to patients and family members (Monsieurs et al., 2015). Asking questions and receiving information about the cardiac arrest and death is important for bereaved family members (Carlsson et al., 2020; Yeates et al., 2013). Bereavement follow-up is common in palliative care and has been shown to facilitate the grief process (Milberg et al., 2008), and it is also an opportunity for healthcare professionals to assess the need for further support. Persons suffering from prolonged grief and psychological health problems need relevant interventions in relation to their problems (Shear, 2012), and further research should explore bereaved family members’ support needs when losing a close person due to cardiac arrest.

### Methodological Considerations 

This study has some limitations that need to be considered. Most importantly, the drop-out rate was high, which resulted in a restricted sample size. Due to the vulnerable situation among family members, problems with drop-out are common in bereavement studies (Stroebe et al., 2003), and studies of sudden death indicate even more problems with recruitment and dropouts (Kõlves et al., 2020). Although the drop-out analysis did not show any significant differences between the participants and the non-participants concerning sex or relation to the deceased, selection bias cannot be excluded. Common reasons not to participate in bereavement studies are fear that this might increase their grief, others might not want to look back since they have ‘got over it’ (Stroebe et al., 2003). Both types of drop-out can bias the findings, and the results should therefore be generalised with care. Related to the restricted sample size, only descriptive statistics and standard inferential statistical methods that can be applied to small samples were used. Finally, this cross-sectional study only measured symptoms of prolonged grief and self-reported health at six months after the death, and thus more research is needed to increase knowledge about longitudinal variations.

## Conclusion

Sudden death by cardiac arrest has a significant impact on bereaved family members’ grief reactions and health. The relation to the deceased person is of significant importance, and especially spouses seem to be at risk for prolonged grief. Despite the fact that most bereaved family members have their own resources to handle the loss, these findings show the importance of offering bereavement support to those at risk for prolonged grief and health problems.
